# Cannabinoquinones: Synthesis and Biological Profile

**DOI:** 10.3390/biom11070991

**Published:** 2021-07-05

**Authors:** Diego Caprioglio, Daiana Mattoteia, Orazio Taglialatela-Scafati, Eduardo Muñoz, Giovanni Appendino

**Affiliations:** 1 Dipartimento di Scienze del Farmaco, Università del Piemonte Orientale, Largo Donegani 2, 28100 Novara, Italy; diego.caprioglio@uniupo.it (D.C.); daiana.mattoteia@uniupo.it (D.M.); 2 Dipartimento di Farmacia, Università di Napoli Federico II, Via Montesano 49, 80131 Napoli, Italy; scatagli@unina.it; 3 Instituto Maimónides de Investigación Biomédica de Córdoba, 14004 Córdoba, Spain; 4 Departamento de Biología Celular, Fisiología e Inmunología, Universidad de Córdoba, 14071 Córdoba, Spain; 5 Hospital Universitario Reina Sofía, Universidad de Córdoba, 14004 Córdoba, Spain

**Keywords:** cannabinoids, quinones, oxidation, thia-Michael addition, immunomodulation, scleroderma

## Abstract

Neutral cannabinoids are oxidatively unstable and are converted into quinone derivatives by atmospheric- and/or chemical oxidative dearomatization. The study of cannabinoquinones has long been plagued by their lability toward additional oxidative degradation, but full substitution of the quinone ring, as well as the introduction of steric hindrance on the alkyl substituent, have provided sufficient stability for a systematic investigation of their bioactivity and for further clinical development. These studies culminated in the discovery of the aminocannabinoquinone VCE-004.8 (**5**), a compound under phase 2 clinical development with orphan drug status by EMA and FDA for the management of scleroderma. The synthesis and rich chemistry of these compounds will be described, summarizing their biological profile and clinical potential.

## 1. Introduction

**Figure 1:**
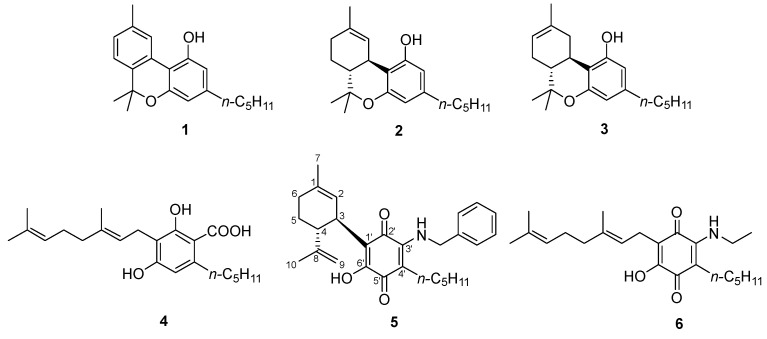
Structure of CBN (**1**), Δ^9^-THC (**2**), Δ^8^-THC (**3**), cannabigerolic acid (CBGA, **4**), VCE-004.8 (**5**) and VCE 003.2 (**6**).

Phytocannabinoids are endogenously produced by *Cannabis sativa* L. in an acidic form, a discovery reported by Krejčí and Šantavý [1] five decades after the seminal purification of cannabinol (CBN, Figure 1, **1**) from hashish in 1899 [2]. Exogenous factors (heat, light, oxygen) are next responsible for the non-enzymatic expansion of the diversity of the native phytocannabinoid pool [3]. Neutral cannabinoids are generated by decarboxylation of their acidic precursors, a reaction dependent on the presence of a mesomerically electron-donating *para*-oxygen atom [4], while atmospheric oxidation can affect both the terpenyl residue, as shown by the aromatization of Δ^9^- and Δ^8^-tetrahydrocannabinols [Δ^9^- and Δ^8^- THC (**2**, **3**, respectively) to cannabinol (**1**) [3], and the resorcinyl core, generating colored quinones [5]. Air oxidation of resorcinyl phytocannabinoids is relatively slow, at least in the plant material and in the solid state, and cannabinoquinones are susceptible to further oxidation and degradation. Compounds of this type have, therefore, only been isolated as trace constituents of *C. sativa* extracts [3]. Nevertheless, they have played an important role in the history of *Cannabis*, since their formation provided the basis for the Beam test, the first forensic color assay for hashish [6], and giving a reddish hue to the high vacuum Cannabis distillates (*red oils*) from which the first phytocannabinoids were isolated and characterized [7]. The lack of narcotic activity of acidic cannabinoids, biogenetically derived from cannabigerolic acid (CBGA, **4**), and of cannabinoquinones, has long been reductively equated to a lack of bioactivity. This and instability issues have combined to delay systematic studies in the area, but significant clinical potential has now emerged for both classes of compounds, and especially cannabinoquinones. Thus, the aminocannabinoquinone VCE-004.8 (**5**) is currently in phase 2 clinical studies with orphan drug status in the EU and USA for the treatment of diffuse cutaneous systemic sclerosis [8]; its analogue VCE-003.2 (**6**) has completed pre-clinical investigation [9], receiving orphan drug designation by the EMA and FDA for the management of Huntington’s disease, and interest in the anticancer activity of cannabinoquinones has been revamped by recent studies [10]. These developments have provided a rationale to review the chemistry and the bioactivity of these compounds, summarizing the early studies as well as the more recent developments.

## 2. Timeline of Studies

**Figure 2:**
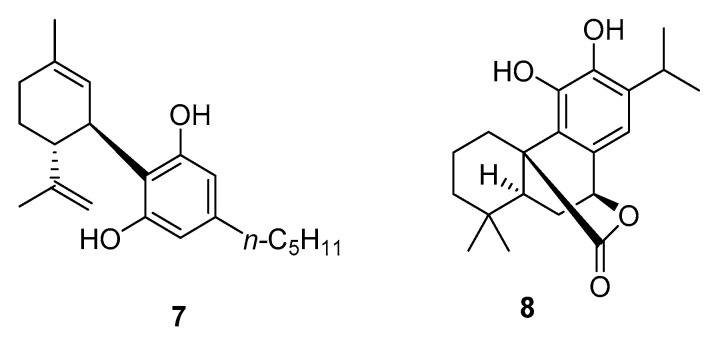
Structure of cannabidiol (CBD, **7**) and carnosol (**8**).

Interest in cannabinoquinones can be traced to the discovery of the first forensic test for hashish. In 1911, Beam reported that ethanolic KOH solutions turn purple–violet upon treatment with hashish [6], a reaction that detects non-narcotic diphenolic phytocannabinoids like cannabidiol (CBD, **7**, Figure 2), and not their mono-etherified analogues like narcotic Δ^9^-tetrahydrocannabinol (Δ^9^-THC, **2**) [11]. This paradoxical situation is reminiscent of the acid color test for opium, that, rather than morphine, actually detects non-narcotic alkaloids from the norrheadine-papaverrubine class, from which, by acidic treatment, deep-colored quaternary isoindolobenzazepines are formed [12]. 

The Beam assay remained the main field test for narcotic cannabis until the Fifties of the past century, when it was replaced in forensic analysis by the more sensitive Duquenois–Levine assay based on the treatment of hashish with vanillin and acetaldehyde under acidic conditions [13]. The Beam assay is, however, more selective, with the curious exception of two common culinary herbs (sage and rosemary), which give a weak positive reaction [13], possibly associated to the presence of the diterpene carnosol (Figure 2, **8**). In recent developments, the Beam and the Duquenois–Levine color assays were complemented by the 4AP (4-aminophenol) assay, which selectively detects Δ^9^-THC (**2**) also in the presence of modest amounts of non-narcotic analogues [14].

**Figure 3:**
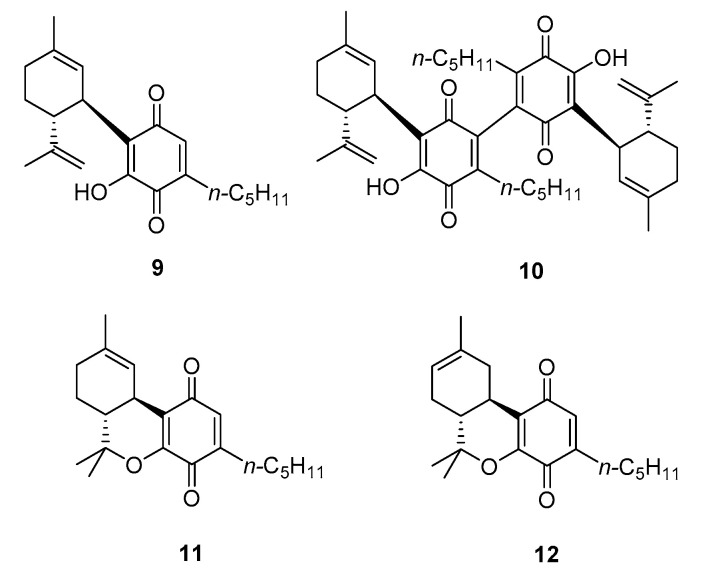
Structure of *p*-CBDQ (HU-331, **9**), its dimer (**10**), *p*-Δ^9^-THCQ (**11**) and *p*-Δ^8^-THCQ (**12**).

The popularity of these field assays has prompted studies aimed at the characterization of the colored compounds formed in these reactions, fostering the discovery of the quinoid form of cannabinoids. Thus, in 1968, the *p*-quinone **9** was obtained by Mechoulam from the treatment of CBD under the alkaline Beam test conditions [11] (Figure 3). The oxidative dimerization of **9** to **10**, and then to further uncharacterized degradation products, was also observed. Spectroscopic considerations ruled out an *o*-quinone structure for **9**, since its UV spectrum did not show the bathochromic shift typical of *o*-quinones, rather absorbing at λ-values typical of *p*-quinones (270 nm, 410 nm) [11]. Δ^9^-THC was stable under these conditions, but its more stable isomer Δ^8^-THC (3) was converted into its corresponding quinone (**12**) upon treatment with peracids [11]. In a later investigation, the *p*-quinone structure assigned to **12** was challenged by NMR studies, and an isomeric *o*-quinone alternative was proposed [15]. Since **12** could be obtained by the acidic treatment of the quinone **9**, the structure of this compound also became questionable.

The original *p*-quinone assignment of 9 and 11 was eventually confirmed by an X-ray study, that debunked as an artefact related to signal overlapping the NOE-based NMR argument on which the structure revision had been suggested [16]. These studies were triggered by the discovery of the selective cytotoxicity of the quinone 9 (HU-331, CBDQ) [16], that fueled a series of studies aimed at elucidating its mechanism of action and toxicity that were published in the early decade of this century [10,17].

**Figure 4:**
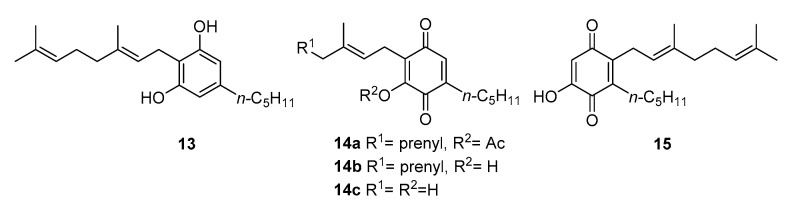
Structure of cannabigerol (CBG, **13**), CBGQ-acetate (**14a**), CBGQ (**14b**), deprenyl-CBGQ (**14c**) and abnormal CBGQ (**15**).

Possibly because of stability issues, these promising studies did not materialize in clinical development, but, after a decade gap, interest in cannabinoquinones was rekindled by the discovery of their selective activation of the peripheral cannabinoid receptor CB_2_ and modulation of the peroxisome-proliferator activator receptor (PPAR)-γ [8,9]. This, and the detection of three cannabigerol (**13**, CBG)-related quinones in marijuana [cannabigeroquinone acetate (**14a**, CBGQ-acetate), CBGQ (**14b**), deprenyl-CBGQ (**14c**), and **15** (abnormal-CBGQ)] [3] (Figure 4), fostered systematic studies aimed at solving the instability issue and at exploiting the clinical potential of these compounds in a non-oncological setting.

## 3. Synthesis and Chemistry of Cannabinoquinoids

### 3.1. Synthesis

Despite its simplicity, the translation of the Beam test of hashish into a reliable synthetic conversion of phytocannabinoids to cannabinoquinones was not straightforward. The first protocol proposed was the aerobic treatment of a resorcinyl phytocannabinoid in a cooled (0 °C) biphasic mixture of petroleum ether and 5% ethanolic KOH [11]. This method had issues of scalability and reproducibility and was plagued by the formation of dimeric quinones (see Section 3.2). Inspired by the Uskokovic hydroxylation of deoxyquinine [18], we found that the oxidation could also be carried out by simply stirring in an open flask a DMSO or THF solution of a resorcinolic phytocannabinoid with sodium hydride or potassium *tert*-butylate. After neutralization with diluted HCl, quinones could be recovered by extraction with petroleum ether-ether 3:1. Under these conditions, dimerization was substantially reduced compared to the biphasic system, but the reaction could not be scaled up, even when air was bubbled into the reaction mixture [5]. The addition of DMSO as a peroxide quencher has been reported to have beneficial effects on the biphasic oxidation, affording a rewarding 50% yield for the oxidation of CBD (**7**) to CBDQ (**9)** when the reaction was carried out in an oxygen atmosphere [10]. 

**Scheme 1:**
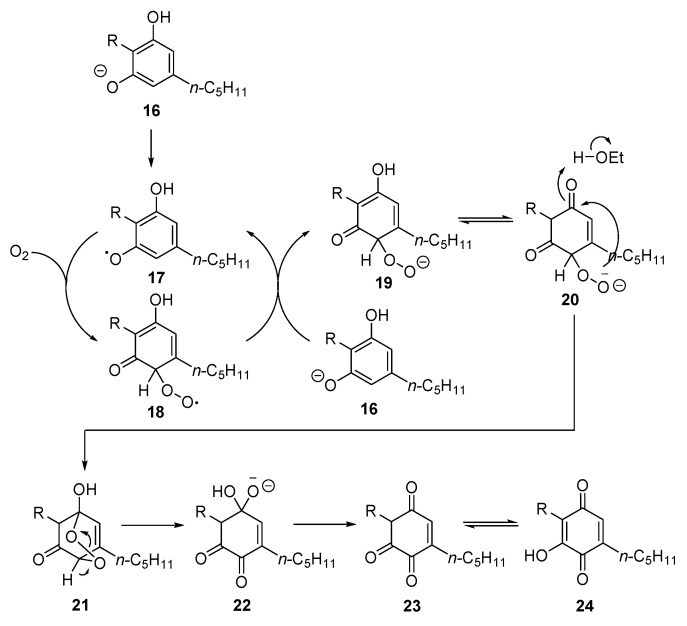
Base-promoted autooxidation of diphenolic cannabinoids to quinones (**23** and **24**) (Beam reactionvia intermediates **16–22** (R = terpenyl).

The Beam reaction presumably involves the generation of an electrophilic radical by the mono-electronic oxidation of a phenolate anion (Scheme 1). This next reacts with dioxygen affording a hydroperoxyl radical, whose reduction to the corresponding anion by a second phenolate ion establishes a reaction loop that propagates the reaction. The final quinone is generated from the hydroperoxyl anion with a mechanism reminiscent of the vitamin K cycle [5]. Thus, after tautomerization of the dienol system to a dicarbonyl form, the hydroperoxyl anion is trapped by the *para*-carbonyl, affording a peroxyhemiacetal. Cleavage of the peroxidic bond is triggered by α-deprotonation, generating an *o*-quinone that eventually tautomerizes to a more stable and intramolecularly hydrogen-bonded *p*-quinone. The mechanism outlined in Scheme 1 requires two free phenolic hydroxyls, the first one to generate a phenolic radical, and the second one to trap, after tautomerization, the hydroperoxyl anion. This auto-oxidative mechanism is in accordance with the delaying effect of antioxidants on the reaction as well as with the stability of monophenolic phytocannabinoids like Δ^9^-THC (**2**), under these reaction conditions. 

**Figure 5:**
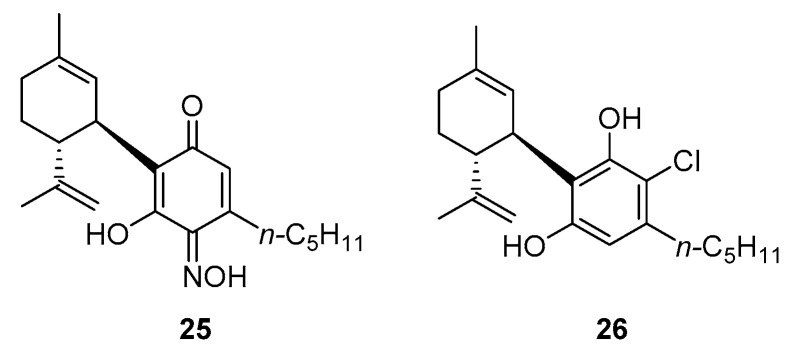
Structure of hydroxyiminodienone (**25**) and chlororesorcinol (**26**), resulting from treatment of CBD (**7**) with the Takehira reagent.

Both mono- and diphenolic phytocannabinoids could be oxidized to quinones using metal-based reagents or peracids. Razdan reported the copper (I) catalyzed oxidation of Δ^8^-THC (**3**) to the quinone **12**, wrongly assigned an *o*-quinone structure due to a misleading NMR NOE-experiment, as well as on the mechanistic assumption of a similarity with tyrosinase, a copper enzyme that oxidizes phenols to catechol and next to *o*-quinones [15]. Using CBD (**7**) as a substrate, a variety of metal oxidants was investigated, including Fe (III) (FeCl_3_, K_3_[Fe(CN)_6_], Mn (IV) (MnO_2_), Cr (VI) (CrO_3_, pyridinium chlorochromate. pyridinium dichromate), Ag (I) (Ag_2_O), Cu (I) (CuCl), Cu (II) (CuCl_2_), Ce (IV) [NH_4_Ce(NO_3_)_5_] under both catalytic or stoichiometric aerobic conditions [5]. All reagents could generate the quinone **9**, but yields were erratic and not better than the one of the autooxidation under biphasic basic conditions. Similar observations were done with peroxidic oxidants [peracids, *tert*-butyl hydroperoxide (TBHP), basic H_2_O_2_], while the combination of hydroxylamine and CuCl_2_ (Takehira reagent) gave a mixture of the hydroxyiminodienone **25** and the chlororesorcinol **26** (Figure 5) [5]. Taken together, these observations show that, although straightforward in terms of color development, the conversion of resorcinyl-phytocannabinoids into hydroxyquinones is not trivial to translate from a color assay into a synthetic maneuver.

**Scheme 2:**
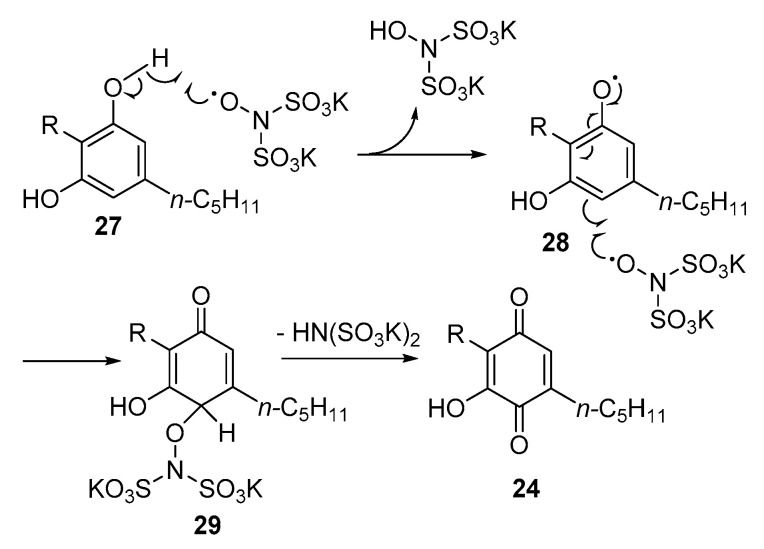
Proposed mechanism for Fremy’s salt oxidation or resorcinolic phytocannabinoids (**27**) to *p*-quinones (**24**) via intermediates **28** and **29** (R = terpenyl).

An alternative radical-mediated oxidative dearomatization of phytocannabinoids to cannabinoquinones was reported using Fremy’s salt (potassium nitrosodisulfonate), a stable radical dianion (Scheme 2). The *p*-selectivity of the oxidation is the result of orbital considerations, namely the larger SOMO coefficients of the phenoxy radical at the *p*-carbon [19]. At the sub-millimolar scale, Fremy’s salt could oxidize CBD (**7**) to CBDQ (**9**) in >90% yield (Scheme 2). However, the hazardous nature of the reagent makes it unsuitable for larger scale preparations. The electrochemical oxidation of cannabinoids to *p*-quinones was investigated by Garg in connection with the development of a possible marijuana breath analyzer [20]. Under carefully controlled conditions (graphite electrodes, NBu_4_BF_4_ electrolyte, 60 s polarity alternation), Δ^9^-THC (**2**) gave the *p*-quinone **11** in a rewarding 67% yield [20].

**Scheme 3:**
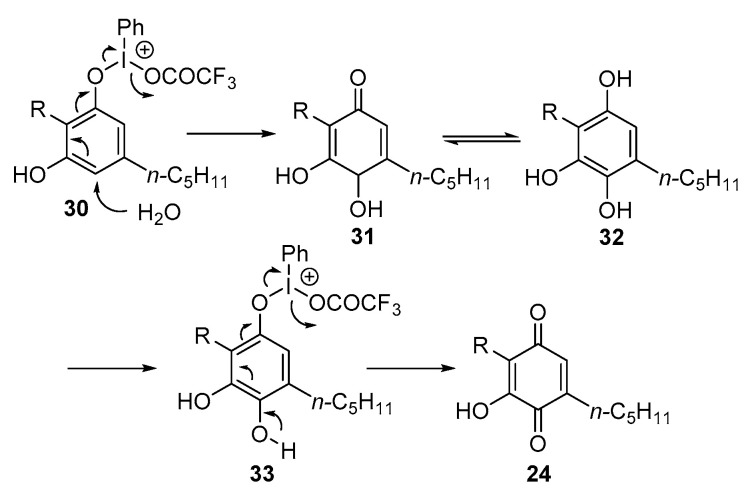
Oxidation of resorcinolic phytocannabinoids (**30**) to cannabinoquinones (**24**) with the aryloxyiodonium (III) oxidant PIFA via the intermediates **31–33** (R = terpenyl).

The formation of cannabinoquinones under non-radical conditions was first reported by Mechoulam, who discovered that the λ^3^-periodinane bis(trifluoroacetoxy)iodobenzene (BTIP, PIFA) could convert Δ^8^-THC (3) into the quinone **12** [16]. The reaction involves formation of an iodonium (III) intermediate, that next undergoes nucleophilic attack by an external water molecule to the *para*-position, less encumbered compared to the *ortho*-positions. Elimination of iodobenzene eventually generates a *p*-quinone in yields between 31% and 50% (Scheme 3).

**Scheme 4:**
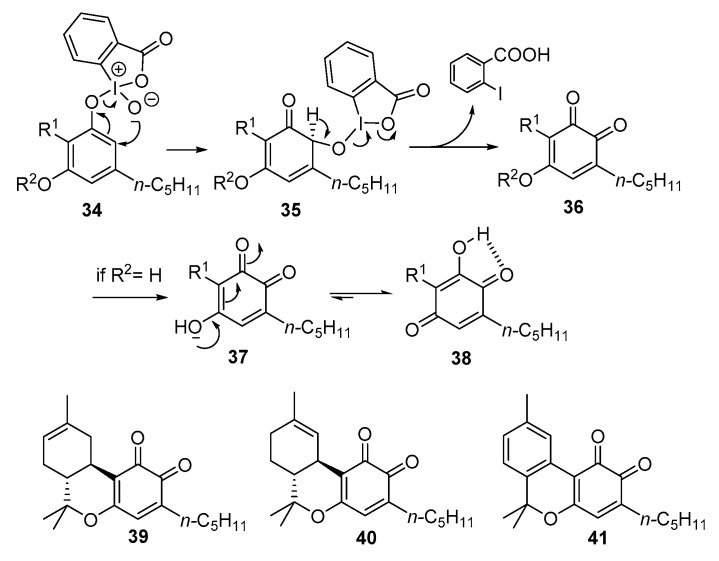
Oxidation of resorcinolic phytocannabinoids (**34**) with the aryloxyiodonium (V) oxidant IBX to quinones **36–38** via the intermediates **35** (R^1^ = terpenyl) and quinones **39–41**.

Superior yields (37–61%) were obtained with the λ^5^-iodane iodoxybenzoic acid, used as its non-explosive formulation SIBX [5]. The reaction involves the initial formation of an iodonium(V) intermediate, that then undergoes a [2,3]-sigmatropic rearrangement of the iodine–oxygen bond, followed β-elimination of 2-iodobenzoic acid to an *o*-quinone. With diphenolic phytocannabinoids, prototropic equilibration then takes place, affording the corresponding and more stable *p*-quinones (Scheme 4). Monophenolic phytocannabinoids like Δ^9^- and Δ^8^-THC (**2** and **3**, respectively) and CBN (**1**), where this tautomerization cannot take place, afforded the corresponding *o*-quinones (**39**–**41**). 

**Scheme 5:**
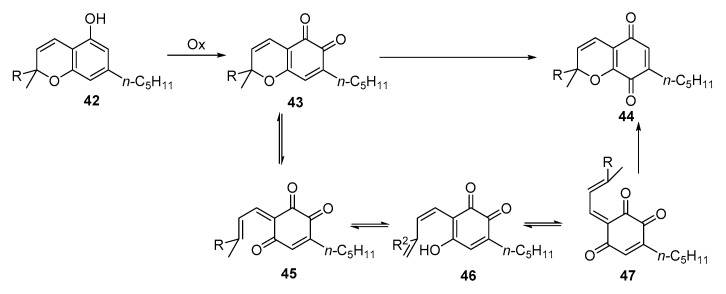
Oxidation of CBC (**42**) to *o*-CBCQ (**43**, and its valence tautomerization to *p*-CBCQ (**44**) via the intermediates **45–47**.

Unexpectedly, oxidation of the monophenolic phytocannabinoid cannabichromene (CBC, **42**) afforded a *p*-quinone (**44**) also with SIBX [21], presumably because of electrocyclic opening of the chromene ring of an originally generated *o*-quinone (**43**) to a cyclohexentrione (**44**), that by a series of keto-enolic tautomerization steps undergoes electrocyclizative aromatization to a *p*-quinone (Scheme 5) [22].

Acidic cannabinoids are stable under Beam-conditions, and did not afford quinones upon reaction with SIBX, that, just like the Beam biphasic system [9], could oxidize 2-alkyl substituted cannabinoids to their corresponding quinones. Taken together, the results of these synthetic studies have established iodanes as the oxidants of choice for the generation of cannabinoquinones from phytocannabinoids. The λ^5^-iodane SIBX can be used for diphenolic substrates, while the λ^3^-iodane PIFA is required for monophenolic phytocannabinoids, with the notable exception of chromenic substrates, where valence equilibration converts *o*-quinones into *p*-quinones [21]. The dimerization that plagues the autoxodidative reactions is not observed with iodane oxidants, and any potential issue of metal retention by chelation is also prevented. The iodinane oxidation of resorcinolic cannabinoids could be scaled-up to gram-levels [5], and proved suitable for the GMP-production of the amounts of VCE-004.8 required for the phase 1 clinical studies [23].

### 3.2. Reactivity

#### 3.2.1. Dimerization

**Figure 6:**
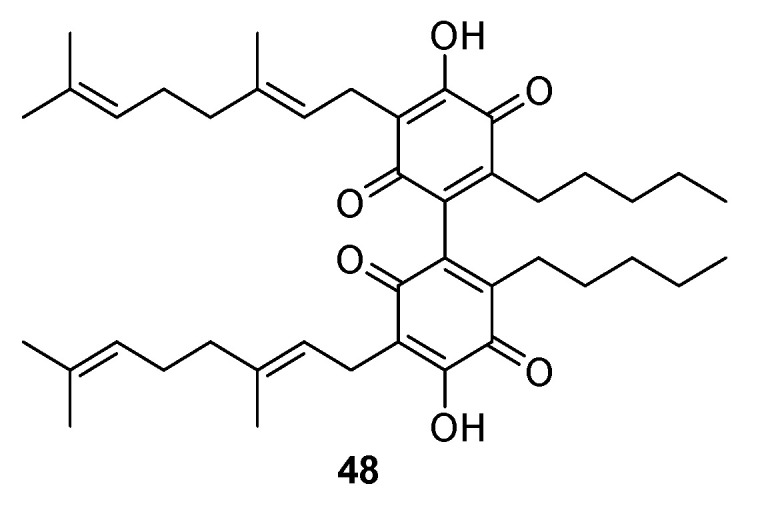
Structure of cannabigerol quinoid dimer (**48**).

Under conditions of single-electron oxidation (auto-oxidation or metal-promoted processes), cannabinoquinones are oxidatively dimerized by 3′,3′-coupling, generating axially asymmetric dimers, enantiomeric or diastereomeric, depending on the chirality of the starting cannabinoid [11]. Dimeric cannabinoquinones are considerably less stable than their corresponding monomeric precursors, and can be stored only in frozen benzene or frozen DMSO [5]. No definite compound could be characterized from their degradation, that was rapid (days) both in solution and in the solid state. Dimerization could be prevented by the introduction of a substituent at C-2′, by steric hindrance on the C-3 alkyl substituent, or by alkylation of the enolic proton. The racemic dimeric quinone (**48**) from cannabigerol (CBG, Figure 6, **13**) was resolved by chiral chromatography, but both enantiomers turned out to be inactive in assays of PPAR-γ modulation [5]. The diastereomeric mixture of CBDQ dimers **10** was also devoid of cytotoxicity [10]. Because of their instability and lack of bioactivity, at least with these endpoints, the biological profile of dimeric cannabinoquinones was not further investigated.

#### 3.2.2. *O*-Methylation

**Scheme 6:**
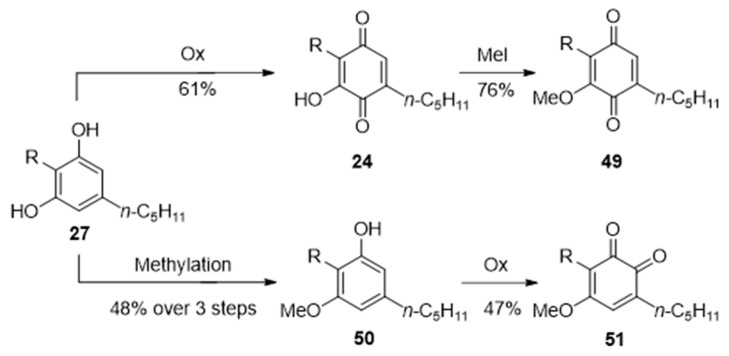
Regiodivergent formation of isomeric *O*-methylcannabinoquinones (**49, 51**) from resorcinolic phytocannabinoids. (**27**, R = terpenyl) via, respectively, the quinones **24** or the resorcinols **50**. Yields for R = Δ^1,8^-3-*p*-menthadienyl, that is, cannabidiol (compound 7)).

The *ortho, para*-prototropic equilibration of enolic cannabinoquinones can be frozen by *O*-methylation, a maneuver that, depending on the sequence of oxidation and methylation, can afford regioisomeric methylated cannabinoquinones from the same starting phytocannabinoid (Scheme 6). Thus, SIBX oxidation of resorcinolic phytocannabinoids affords their *p*-quinone derivatives, next converted by methylation into their corresponding non-equilibrating *O*-methyl derivatives [21]. Conversely, if methylation is carried our first, due to the impossibility of post-oxidative tautomeric equilibration, *ortho*-quinones are instead obtained [21]. These isomeric quinones have distinct spectroscopic features (See Section 4) and can also be distinguished visually due to the more intense and red-shifted hue of the *o*-isomers, clearly less stable compared to their corresponding *p*-isomers [21]. Methylation of cannabinoquinones was associated with significant activity on the Nrf2-Bach1 axis (See Section 5.3).

#### 3.2.3. Thia- and Aza-Michael Addition

**Scheme 7:**
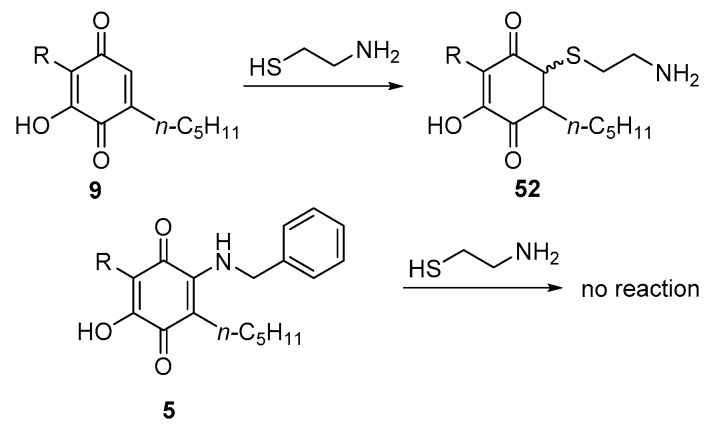
Michael reactivity towards thiols of CBDQ (**9**) and VCE-004.8 (**5**) via the intermediacy of the sulphide **52** (R = terpenyl).

Cannabinoquinones are highly electrophilic compounds and undergo non-transient thia-Michael addition when assayed in the cysteamine assay (Scheme 7) [24]. With 2-substituted compounds, the assay was complicated by the interaction of the amino group of cysteamine with the quinone carbonyl, resulting in a change of the DMSO spectrum upon cysteamine addition, presumably due to the transient Schiff base formation. This resulted in chemical shift changes, with no evidence, however, of thiol addition [24]. Phase switch to CDCl_3_ or extraction with hexane regenerated the starting compound, showing that the introduction of a substituent at the 2-position quenches Michael reactivity, an observation of relevance for the bioactivity profile of cannabinoquinones (see Section 5.1).

The aza-Michael addition of primary amines was at the basis of the development of compounds from the VCE-series [8,9]. Cannabinoquinones react with primary amine to afford transient 1,4-adducts that spontaneously undergo dehydrogenation to the corresponding vinylogous amides by reaction with atmospheric oxygen. Since these amine adducts have a different reactivity profile and bioactivity compared to their starting quinones, we have named these compounds cannabinoquinones. The reaction could be carried out with a diversity of primary amines, while secondary amines were unreactive. Moreover, cannabinoquinones from the α,α-dimethylheptyl series were unreactive due to increased steric hindrance [23]. Thus, substitution at C-2 or at the benzyl carbon of the alkyl substituent could solve the stability issues associated to oxidative dimerization, also quenching thia-Michael reactivity. The tandem addition-dehydrogenation reaction with primary amines gave better yields in polar solvents, an observation that cannot be explained in terms of oxygen solubility, since this gas, responsible for the dehydrogenation of the adducts, is more soluble in toluene and dichloromethane, poor performers, compared to alcohols, ethyl acetate or acetone, that gave the higher yields. The aza-Michael addition was unsuccessful with *O*-alkylated cannabinoquinones like **11**, **12** or **44**, suggesting that the engagement of the C-4′-carbonyl in an intramolecular hydrogen bonding is critical to lower the LUMO of the 2′,3′-enone system and promote the addition step. The synthesis of aminocannabinoquinones could also be telescoped into a one-step protocol by treating the filtered crude reaction mixture from the SIBX oxidation with an excess of amine. Purification of the final product was, however, laborious, and overall yields were lower [23].

## 4. Spectroscopic Properties of Cannabinoquinones

**Figure 7:**
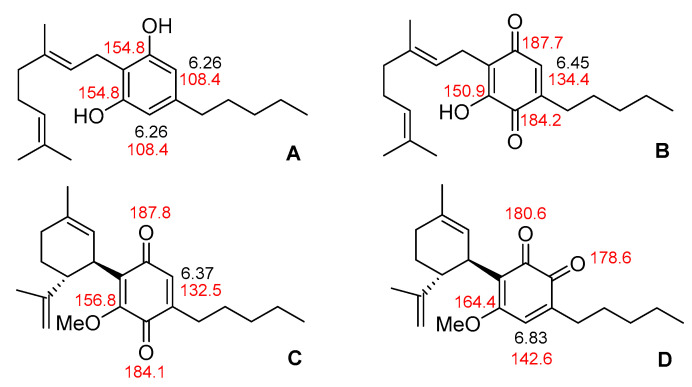
Top: Comparison of selected ^1^H (black) and ^13^C (red) NMR resonances of a phytocannabinoid (CBG, **A**) with those of the corresponding quinone (**B**). Bottom: Comparison of selected ^1^H (black) and ^13^C (red) NMR resonances of *O*-methyl-*p*-CBDQ (**C**) and *O*-methyl-*o*-CBDQ (**D**).

Parallel to the color development, NMR spectroscopy can be an efficient system to detect and monitor the conversion of phytocannabinoids into the corresponding monomeric quinones. ^1^H NMR spectra of *p*-cannabinoquinones are characterized by the obvious loss of one of the two resorcinyl protons, with the remaining signal experiencing a downfield shift of ab. 0.2–0.3 ppm (Figure 7) [5]. The hallmark of *p*-cannabinoquinones ^13^C NMR spectra is the presence of the two carbonyl resonances around δ_C_ 180–190 ppm. The third oxygen-bearing carbon of the same ring resonates at ~δ_C_ 150 ppm, while the single methine experiences a marked downfield shift to ~δ_C_ 130 ppm [5,21]. 

**Figure 8:**
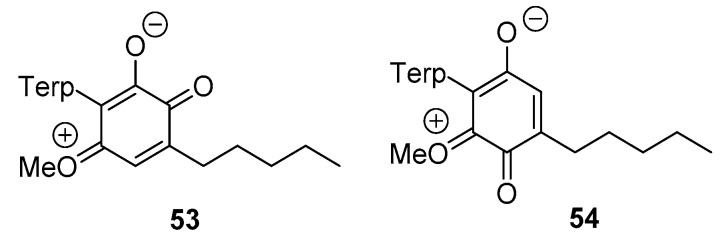
Structure of the zwitterionic resonance for *O*-methyl-*ortho*-cannabinoquinones (**53**) and *O*-methyl-*para*-cannabinoquinones (**54**).

As described in Section 3.2.2, a different order in the methylation/oxidation sequence can provide alternative *ortho*- and *para*-*O*-methyl cannabinoquinones, providing the opportunity of a *vis-à-vis* spectroscopic comparison between isomeric quinoids. As shown in Figure 7, the single proton signal of the quinone ring is markedly downfield shifted (Δδ ab. 0.4 ppm) in *o*-cannabinoquinones and other diagnostic differences are also present in the ^13^C NMR spectra. Indeed, the *ortho*- isomer can be easily identified, since the couple of carbonyl signals resonates at significantly lower ppm values compared to the *para*- one (δ_C_ 175–180 vs. δ_C_ 185–190) [22]. Both this high-field shift and the higher resonance values of the -OMe-bearing carbon can be explained by considering that an *ortho*-quinone carbonyl is a better electron sink than a *para*-quinone carbonyl for the mesomeric delocalization of the lone pair of the methoxy oxygen. Thus, due to a more extended conjugation, the contribution of the zwitterionic resonance form that includes a methoxonium ion is larger in *ortho*-cannabinoquinones compared to *para*-cannabinoquinones (Figure 8).

## 5. Bioactivity Studies

Quinones are formed spontaneously in a solution of resorcinolic phytocannabinoids [10]. In turn, cannabinoquinones rapidly degrade in solution with the development of a purple color [10]. DMSO solutions of CBDQ (HU-331, **7**) have been reported to lose 50% of their titer in 2 days at 20 °C [19]. In bioactivity studies, care should therefore be taken to avoid contamination of resorcinolic phytocannabinoids with cannabinoquinones, and to prevent the degradation of cannabinoquinone solutions. 

### 5.1. Anticancer Activity

Interest for cannabinoquinones was spurred by the discovery of the anticancer activity of CBDQ (HU-331), which in cellular models could inhibit, at low μM concentrations, the proliferation of several cancer cell lines [16]. In later studies, potent inhibition of vascular cell proliferation involved in angiogenesis was also observed [25], and topoisomerase II in its two isoforms (TOPO2A and TOPO2B) was identified as a possible anticancer target [26,27]. This profile of activity is the hallmark of anthracyclines (doxorubicin, daunorubicin) and deoxypodophyllotoxin-derived (etoposide, teniposide) anticancer agents, but, remarkably, when comparatively investigated with doxorubicin in murine xenografts, the activity of CBDQ was not associated with cardiac toxicity or to myelosuppression, the typical side-effects of topoisomerase poisons, even though CBDQ outperformed doxorubicin in terms of potency [28]. Remarkably, the activity of CBDQ was not associated to the generation of haeme-mediated oxygen radicals, to intercalation, or to weight loss. This is in sharp contrast to other results showing that HU331 is electrophilic, induces ROS and disruption of mitochondrial transmembrane potential and activates the Nrf2 pathway in Jurkat cells [8]. These observations suggested that, even if topoisomerase II were the single major target of CBDQ, its mechanism of interaction is basically distinct from the one of topoisomerase poisons. Thus, CBDQ has been suggested to target the ATPase domain of the enzyme by covalently binding two reacting cysteines (cys-392 and cys-405) [19]. This blocks the ability of the enzyme to relax supercoiled DNA without causing strand breaks [29]. A structure-activity study on CBDQ using cytotoxicity on Raji and Jurkat cell lines showed results indicative of a specific and not a class-type activity. Thus, both monocyclic (CBGQ, **14b**) and tricyclic (Δ^9^-THCQ, **11**) cannabinoquinones were significantly less active than CBDQ (HU-331, 9), while a change of the substitution pattern of the quinone motif to compounds from the abnormal series, removal of the isopropenyl group from the *p*-menthyl moiety as well as the changes detrimental for thia-Michael reactivity (substitution at C-2′, substitution at the benzyl position of the pentyl residue) reduced activity. The observation that *O*-methylation was tolerated, despite its effect on Michael reactivity is surprising [10], since a study on fully synthetic analogues of HU-331 showed that *O*-methylation was detrimental for both topoisomerase inhibition and cytotoxicity [19]. Interestingly, homochiral and racemic CBDQ (HU-331) were equipotent in both assays [19]. 

Taken together, these studies show that cannabinoquinones are interesting structures for the development of novel anticancer agents, but the stability in solution of the lead compound of the class (CBDQ, HU-331, **9**) seems too limited for further developments. In addition, the mechanism of activity needs additional investigation, while the pharmacokinetic profile of these compounds is still unknown.

### 5.2. Modulation of Liver Microsome Activity

Formation of CBDQ (**9**) was observed during the murine metabolism of CBD (**7**) [30,31,32,33] and could potentially play a role in liver damage observed with high dosages of CBD in rodents [34]. Liver damage could be associated with thiol depletion, to the generation of reactive oxygen species, to the deactivation of catalase and superoxide dismutase, or to a combination of these mechanisms [30,31,32,33]. CBDQ potently inhibited murine microsomal CYP450 hydroxylase, as well as *O*- and *N*-demethylase activity [31]. This profile of inhibition was also shown by CBD, but to a much lesser extent and with a distinct mechanism that required an NADPH-generating system, either cytoplasmic (pentose phosphate shuttle) or mitochondrial. Conversely, CBDQ could directly affect the microsomal system, presumably because of covalent binding to reacting cysteines [31,32]. This view was supported by the observation that glutathione and cysteine attenuated the activity of CBDQ on CYP450 activity [31], and by the detection, by mass spectrometry, of a covalent adduct of CBDQ with CYP450 3A11 [32]. The translation of these observations on a human setting is unclear. CBDQ is not a major human metabolite of CBD [34], but liver toxicity remains an unsettled issue for the clinical development of thiophilic cannabinoquinones.

### 5.3. Neuroprotective and Immunomodulatory Activity 

Interest in cannabinoquinones in the neuro-immunological area was spurred by considerations of the biological profile of CBD (**7**). This compound has a multitude of low-affinity targets that includes GPCR, ion channels, enzymes, and transcription factors of critical relevance for neuroprotection and immunity [35]. The twisted bicyclic structural element of CBD seems to have an inherent, broadly tuned complementarity for biological surfaces, possibly expressed by different torsion angles along the methyl-aryl atropoisomeric axis, unable, however, to engage the compound in tight intermolecular interactions. In this context, the increased polarization associated to the resorcinol-to-hydroxyquinone oxidation is expected to strengthen electrostatic and hydrogen bonding interactions with macromolecular targets, additionally increasing biological “stickiness” by metal chelation, covalent reactivity via Michael addition and involvement in redox cycles. This strategy was confirmed by comparison of the PPAR-γ modulation activity of cannabidiol (CBD) and its corresponding quinone (CBDQ). While the former showed only marginal activity in the assay (EC_50_ > 25 μM), the latter was endowed with potent activity (EC_50_ = 10.5 μM), further increased by one order of magnitude by the aza-Michael adducts [8]. These observations were duplicated with the quinones of cannabigerol (CBG, **13**) and CBC (**42**) [9], suggesting a class-specific trend, that led to extensive studies and to the selection of two candidates for systematic studies.

The CBG-derived amino-hydroxyquinone **6** (VCE-003.2) showed potent agonistic activity at PPAR-γ (IC_50_ = 1.2 μM), binding both the canonical and the alternative binding sites of the transcription factor, and lacking activity on cannabinoid receptors [36]. In BV2 mice microglia- and astrocytes cells, **6** could inhibit the secretion of various soluble pro-inflammatory mediators (IL-6, TNF-α, IL-1b) at one-digit μM concentration, reducing the expression of iNOS in BV2 microglia cells in a way that is sensitive to PPAR-γ (GW9662) antagonists [36]. When orally or intraperitoneally administered, **6** showed beneficial effects in murine models of Huntington’s disease [9,37], Parkinson’s disease [36,38], and amyotrophic lateral sclerosis [39]. Compound **6**, formulated as EHP-102, has completed preclinical development and is entering a phase 1 study for Huntington’s disease and Parkinson’s disease, with orphan drug status in the EU and USA for the former condition [40]. 

Within the aminoquinones derived from CBD (**7**), the benzylamine adduct **5** emerged as a powerful activator of the PPAR-γ transcriptional activity [8] and, of the hypoxia inducible factor (HIF-1) pathway, an activity resulting from inhibition of prolyl hydroxylases [41]. This aminoquinone **5** (VCE-004.8) was neutral at CB_1_ but behaved as an activator at CB_2_ [8], and was a partial PPAR-γ activator, devoid of adipogenic activity. In a murine model of metabolic syndrome (high-fat diet), **5** could reduce the metabolic disruption and inflammation associated to this condition, promoting weight loss, and reducing total fat mass, adipocyte volume, and plasma triglycerides [42]. Remarkably, it could also increase FGF21 mRNA expression in white and brown adipose cells, as well as in a BAT cell, significantly ameliorating glucose tolerance, reducing leptin levels and increasing adiponectin and incretins (GLP-1, GIP) levels [42]. VCE-004.8 (**5**) showed anti-fibrotic activity in a murine scleroderma model as well as in different multiple sclerosis models, preventing immune cell infiltration, demyelination, and axonal damage [43]. Its oral formulation (EHP-101) has completed a Phase 1 study on over 100 healthy volunteers, demonstrating excellent safety and tolerability, and is proceeding now, under orphan drug status by EMA in Europe and FDA in USA, to a Phase 2 clinical study for the management of scleroderma [44].

## 6. Conclusions

Cannabinoquinones are “programmed “natural products, downstream to enzymatically produced acidic phytocannabinoids by spontaneous decarboxylation and air oxidation of the resorcinyl core [3]. These structural changes have a dramatic effect on their biological profile, with a focus on cytotoxicity and PPAR-**γ**-modulating properties. These features could be dissected by the introduction of a substituent on the quinone ring, either before oxidation, at the stage of neutral phytocannabinoids (2‘-alkyl cannabinoquinones), or by aza-Michael addition and air dehydrogenation (2′-amino cannabinoquinones). The vinylogouos amides resulting from the addition of primary amines to cannabinoquinones (cannabinoquinoids) are stable compounds, and qualify for pharmaceutical development, as exemplified by VCE-004.8 (**5**), the benzylamine adduct of CBDQ (**9**), and VCE-003.2 (**5**), the ethylamine adduct of CBGQ (**14b**).

## Data Availability

Not applicable.
